# Antagonizing CDK8 Sensitizes Colorectal Cancer to Radiation Through Potentiating the Transcription of e2f1 Target Gene apaf1

**DOI:** 10.3389/fcell.2020.00408

**Published:** 2020-06-12

**Authors:** Bin Chen, Pengbo Wen, Guanshuo Hu, Yang Gao, Xiaojing Qi, Kaili Zhu, Shaopeng Chen, Lijun Wu, An Xu, Guoping Zhao

**Affiliations:** ^1^Key Laboratory of High Magnetic Field and Ion Beam Physical Biology, Anhui Province Key Laboratory of Environmental Toxicology and Pollution Control Technology, Hefei Institutes of Physical Science, Chinese Academy of Sciences, Hefei, China; ^2^University of Science and Technology of China, Hefei, China

**Keywords:** CDK8, transcriptional regulation, e2f1, apaf1, radiotherapy, apoptosis

## Abstract

Radiotherapy is an essential curative treatment modality for colorectal cancer. Apoptosis is the major mechanism of IR-induced cell death and aberrant apoptotic signaling results in radioresistance, which is a hallmark of most, perhaps all, types of human cancers. Potentiating the induction of apoptosis is an emerging strategy for cancer radiotherapy. Here, we determined that targeting CDK8 selectively radiosensitized colorectal cancer through the mitochondria-dependent intrinsic apoptotic signaling, which was mediated through the induction of the transcription of apaf1 that was e2f1- and not p53-dependent. Importantly, the enhanced transcriptional activity of e2f1 was dependent on the kinase activity of CDK8 itself and not on the assembling of the mediator complex. In addition, clinical inhibitor, and *in vivo* studies confirmed the radiosensitizing effect of CDK8. Our results provide a new targeting strategy to improve the radiotherapy of CRC.

## Introduction

Colorectal cancer (CRC) is the third most commonly diagnosed cancer and the second most frequent cause of cancer-related deaths (Roberts et al., 2018), over 1.8 million new CRC cases and 880,000 CRC-associated deaths were reported in Bray et al. (2018). Clinically, surgical resection is considered as the main therapeutic modality for CRC, and radiotherapy is considered as an effective treatment option after surgery (Mishra et al., 2013). However, radioresistance is a major cause of treatment failure, which results in incomplete cure, recurrence, and metastasis (Pal et al., 2018; Xiao et al., 2018; Wu et al., 2019). Therefore, appropriate strategies to improve the radiosensitivity of CRC are urgently needed.

Cyclin dependent kinase 8 (CDK8), a nuclear serine threonine kinase, has come under focus owing to its central role in transcription. In association with the mediator complex, it acts as a molecular bridge between transcription factors, chromatin modifiers, promoters, enhancers and RNA Polymerase II (Liu et al., 2004; Kohler et al., 2019; Klatt et al., 2020). Accumulating evidence suggests an underlying role for the CDK8 module in the Wnt, HIF1α, NFκB, e2f1, and p53 pathways, among others (Donner et al., 2007; Galbraith et al., 2013; Birkenheuer et al., 2015; Rzymski et al., 2015; Chen et al., 2017). As most of these CDK8-dependent pathways are altered in cancer, it is not surprising that CDK8 has been proposed to contribute to tumor development. Notably, CDK8 was first linked to cancer when it was identified as an oncogene that is frequently amplified or overexpressed in CRC (Firestein et al., 2008). Subsequently, CDK8 was found to be amplified in 47% of 123 CRC patient samples in a study and cohort studies revealed a negative correlation between CDK8 expression and the survival of CRC patients (Firestein et al., 2010). In addition, increased level of CDK8 was later found in advanced CRC stages III and IV, suggesting that CDK8 contributes to the progression of colorectal adenoma to carcinoma (Seo et al., 2010). Importantly, it was confirmed that CDK8 has proto-oncogenic effects in CRC as it interacts with the Wnt pathway to enhance the transcriptional activity of β-catenin (Zhao et al., 2013). These observations suggest that CDK8 may play an important role in colorectal carcinogenesis. However, it is not clear whether CDK8 participates in the IR response or affects CRC radiosensitivity.

IR causes DNA damage that activates the p53 and e2f1 pathways in concert or independently to repair the damage or induce apoptosis (Udayakumar et al., 2010). These two pathways induce cell-cycle arrest to repair DNA damage. Depending on the extent of the repair level, the repair process can result in three different cellular outcomes: efficient repair that promotes survival, inefficient repair that leads to survival of gnomically unstable cancer cells, and induction of apoptosis if the damage is severe and irreparable (Sirbu and Cortez, 2013). By altering the functions of p53 and e2f1 through truncation, such that the regions responsible for DNA damage repair are eliminated, efficient killing of tumor cells can be achieved when combined with other therapeutic modalities, such as chemotherapy and IR therapy (Udayakumar et al., 2010). Abrogating the DNA repair function and concomitantly enhancing the proapoptotic functions of p53 and e2f1 represents a novel strategy with clinical potential (Polager and Ginsberg, 2009). Notably, CDK8 has been described as a key regulator that positively or negatively affects the transcription of p53 and e2f1. One study showed that CDK8 was recruited to the p21 promoter in response to specific stress and reported CDK8 to be a stimulus specific positive co-regulator of p53 target genes (Donner et al., 2007). In contrast, another study reported that CDK8 down-regulates the transcriptional activity of e2f1 by phosphorylation, which relieves the repression of e2f1 on β-catenin/TCF pathway, and inhibits e2f1-induced apoptosis in CRC cells (Morris et al., 2008). Given the potent effect of CDK8 in the transcriptional regulation of p53 and e2f1, it is of great interest to target CDK8 in cancer therapy.

In this study, we mainly explored the transcriptional regulation of CDK8 in CRC after IR. We elucidated that targeting CDK8 sensitizes CRC to IR both *in vitro* and *in vivo* through potentiating transcription of e2f1 target gene apaf1. Our study revealed that the IR-induced intrinsic apoptosis in CDK8 knockdown cells was dependent on e2f1 but not p53. Further, the inhibition of e2f1 transcriptional activity by CDK8 was dependent on the kinase activity of CDK8 itself and not on the assembling of the mediator complex. These results provide convincing evidence that CDK8 serves as a promising target to radiosensitize CRC to therapy.

## Materials and Methods

### Reagents and Antibodies

Propidium iodide (PI) were obtained from Invitrogen (Shanghai, China). Primers for quantitative real-time PCR and ChIP analysis were purchased from GENEWIZ (Suzhou, China). Transcriptone-step gDNA removal and cDNA synthesis supermix kit was purchased from Transgen Biotech (Beijing, China). SuperReal PreMix (SYBR GREEN) was purchased from Qiagen (Shanghai, China). The siRNA were purchased from GenePharma (Shanghai, China) and siRNA transfection was carried out using Lipofectamine 2000 (Thermo Fishier, Carlsbad, CA, United States). pLKO.1 plasmid expressing CDK8 shRNA was purchased from GENEWIZ (Suzhou, China). The siRNA sequences and shRNA sequences are listed in Supplementary Table S1. ChIP was performed using SimpleChIP Enzymatic Chromatin IP Kit (Agarose Beads) (Cell Signaling Technology, Danvers, MA, United States). Protein G Sepharose beads was purchased from Beyotime (Shanghai, China). Dimethyl sulfoxide (DMSO) and other chemicals were purchased from Sangon (Shanghai, China). Ponatinib and CCT251545 were purchased from Selleckchem and stored following the manufacturer’s instruction.

The antibodies against p53, e2f1, p-Rpb1 CTD (S2/5), Rpb1 CTD, cleaved caspase 7, cleaved caspase 8, cleaved caspase 9, γH2AX and CDK8 were purchased from Cell Signaling Technology (Danvers, MA, United States). e2f1 (S375) antibody was obtained from Millipore (Temecula, CA, United States). Cleaved caspase 3 antibody was purchased from R&D Systems (Minneapolis, MN, United States). apaf1 antibody was obtained from Proteintech (Wuhan, China) and housekeeping gene β-actin was purchased from ZSGB-BIO (Beijing, China).

### Cell Lines and Human Samples

Human CRC cell lines (HCT116 and LOVO), Human small intestine epithelium cell line (HIEC), Mouse CRC cell line (MC38) and Transformed human embryonic kidney cell line (HEK293T) were purchased from the American Type Culture Collection (ATCC, Manassas, VA, United States). HCT116, HIEC, HEK293T and MC38 cells were maintained in Dulbecco’s modified Eagle’s medium (HyClone, Logan, UT, United States), LOVO cells were maintained in Dulbecco’s modified Eagle’s medium with F12 (HyClone, Logan, UT, United States). All cell lines supplemented with 10% fetal bovine serum (Biological Industries, Israel) and 1% penicillin/streptomycin (Beyotime, Shanghai, China) at 37°C with 5% CO_2_.

Surgically resected tumor and normal part of human colon from six individual patients were obtained through Anhui Medical University (Hefei, China).

### Xenograft Model and Radiation

Stably transfected MC38 cells were inoculated into the subcutaneously in dorsal flank of 4-week-old C57BL/6 wild type mice obtained through Anhui Medical University (Hefei, China). A dosage of 0 Gy and 20 Gy were used to irradiate the mice for 8 days after the injection. Tumor dimensions and volumes (mm^3^) were measured and calculated with calipers every day. In the end, the mice were sacrificed by cervical dislocation on the 18 day after injection and the tumors were harvested. The tumor tissues were fixed in formalin to obtain sections for the TUNEL, H&E and immunohistochemical staining. All animal experiments procedures and uses of clinical samples were performed according to guidelines approved by Committee review of animal experiments in Anhui Medical University.

For both *in vitro* and *in vivo* experiment, the irradiation was carried out in an X-ray irradiator, X-RAD 320 (Precision X-Ray Inc., United States). The indicated radiation dose was determined by the total radiation time basis on the dose rate 4.987 Gy/min controlled by the compute automatically. The equipment was maintained and calibrated every year by the manufacturer to ensure the precision of radiation dose.

### Cell Viability Analysis

The indicated cells were plated in a 35 mm tissue culture plate with 200, 000 cells and cultured for 24 h. After treated or treated with CDK8 shRNA, IR, or CDK8 shRNA plus IR, cells were cultured for 24 h. Then all the cells were harvested in the presence of 2 μg/ml PI. Cell viability was measured by PI exclusion using a flow cytometer (FACScalibur; Beckon Dickinson, San Jose, CA, United States) and normalized as the percentage of the viability of untreated cells.

### Colony Formation Analysis

A total of 800 cells were seeded in 60 mm dish. After treatment, the dishes were incubated for 2 weeks at 37°C in a 5% CO2 incubator for 14 days. Then the dishes were washed with PBS, fixed with a solution containing methanol: acetic acid (V/V = 9:1) for 30 min and subsequently stained with crystal violet for 30 min. The colonies containing more than 50 cells per colony was scored and plotted.

### Immunoprecipitation Assay

Treated cells were washed with cold PBS twice before the addition of lysis RIPA buffer. After centrifugation, Protein G Sepharose Beads were added to the lysate and incubated on a rotator for 30 min at 4°C. The supernatant was transferred to a new tube, after centrifugation at 460 × *g* for 3 min at 4°C. The primary antibody was added to the supernatant and incubated at 4°C for 12 h while gently stirring. Protein G Sepharose Bead slurry was then added to capture the protein complex. After incubation at 4°C for 3 h with gentle agitation, the samples were centrifuged at 1,000 × *g* for 30 s at 4°C. The supernatant was discarded and the pellet was washed with RIPA buffer. Finally, the immunoprecipitates were resuspended in SDS-PAGE loading buffer for Western blot analysis.

### Quantitative Real-Time PCR

The real-time PCR primer sequences for the target genes were summarized in supporting information (Supplementary Table S4). The cycling conditions: 2 min, 95°C for PCR initial heat activation; and 5 s, 95°C for denaturation; and 10 s, 60°C for combined annealing/extension; Number of cycles: 40. mRNA expression was assessed by quantitative real-time PCR on an ABI 7500 FAST Real-Time PCR System (Applied Biosystems, Foster City, CA, United States) using SuperReal PreMix (SYBR Green) in a 48-well plate. RNA levels of the genes of interest were normalized to the act-1 level for comparison and gene expression data were analyzed using the comparative 2^–ΔΔCt^ method. Triplicates for each sample were included for one single reaction.

### Chromatin Immunoprecipitation Analysis

Chromatin immunoprecipitation analysis was conducted according to the manufacturer’s instructions (Cell Signaling Technology, Danvers, MA, United States). Briefly, collected cells were crosslinked with 1% formaldehyde and blocked with glycine. Cells were then washed and digested by micrococcal nuclease. The nuclear pellet was suspended in chromatin immunoprecipitation (ChIP) buffer and sheared by sonication. The sheared chromatin was then incubated with various antibodies, including p53, e2f1, p-Rpb1 CTD (S2/5) and Rpb1 CTD. ChIP-enriched DNA was analyzed by qPCR with ChIP primers (Supplementary Tables S2, S3).

### RNA-seq Analysis

The sequencing was performed by using Illumina HiSeq 4000 (LianChuan Sciences, Hangzhou, China). For each given gene list, pathway and process enrichment analysis has been carried out with the following ontology sources: KEGG Pathway, GO Biological Processes, Reactome Gene Sets, Canonical Pathways and CORUM. All genes in the genome have been used as the enrichment background. Terms with a *p*-value < 0.01, a minimum count of 3, and an enrichment factor >1.5 (the enrichment factor was the ratio between the observed counts and the counts expected by chance) were collected and grouped into clusters based on their membership similarities. More specifically, *p*-values were calculated based on the accumulative hypergeometric distribution, and *q*-values are calculated using the Banjamini-Hochberg procedure to account for multiple testings. Kappa scores were used as the similarity metric when performing hierachical clustering on the enriched terms, and sub-trees with a similarity of >0.3 were considered a cluster. The most statistically significant term within a cluster was chosen to represent the cluster.

### Statistical Analysis

All data are represented as the means plus or minus standard deviations, and all experiments were performed in triplicate at least three times independently. *P* < 0.05 between groups was considered significant. The differences between two groups were analyzed with the Student’s *t* test. One-way analysis of variance (ANOVA) followed by *Post hoc* test was used in multiple groups.

## Results

### Pan Cancer Transcriptome Profiling of CDK8

**FIGURE 1:**
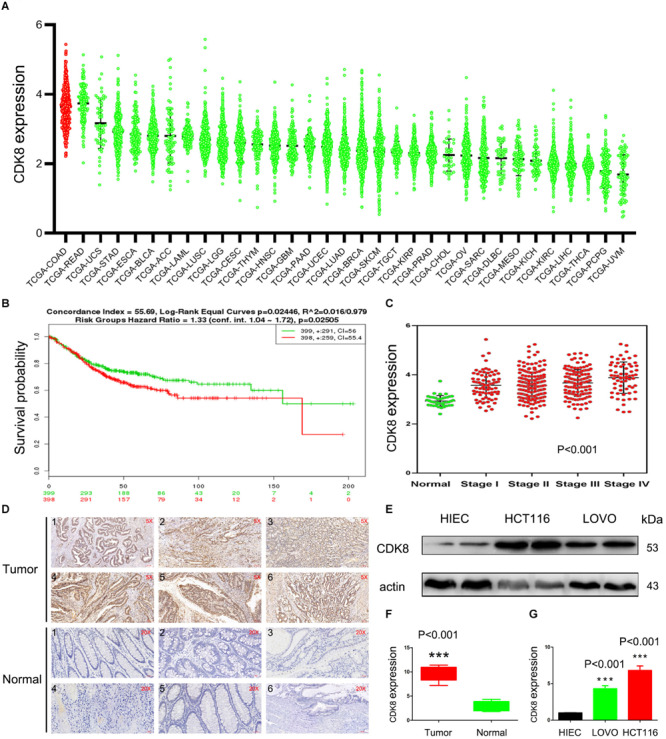
CDK8 Is Overexpressed in CRC. **(A)** Expression of CDK8 in different tumor types in the TCGA database arranged by median. **(B)** Disease free survival of CRC patients with low versus high CDK8 expression. **(C)** Transcriptional expression of CDK8 at different stages of CRC from the TCGA database. **(D)** Immunohistochemical staining for CDK8 expression in representative tissue sections from a large cohort of normal and CRC clinical specimens **(E)** CDK8 protein level was higher in the CRC cell lines, LOVO and HCT116, than in the normal human intestinal epithelial cell line, HIEC, analyzed by western blotting. β-actin served as an internal control. **(F)** Quantitation of the intensity of CDK8 expression in the clinical specimens from panel **(D)** using the IPP software. **(G)** The intensity of CDK8 expression in the CRC cell lines from panel **(E)** were quantified after normalization to β-actin using Image J software. ***p < 0.001. Typical results from three independent experiments are shown. The difference between two groups were analyzed with the Student’s *t* test. One-way analysis of variance (ANOVA) followed by *Post hoc* test was used in multiple groups.

TCGA is an invaluable resource to comprehensively analyze the transcriptome profile of CDK8 across a large spectrum of the most common tumor entities from thousands of patients. In a pan-cancer analysis, the RNA-Seq based expression level of CDK8 across all the 33 tumor entities publicly available at TCGA at the time of analysis for all 10,327 patients was obtained. A scatter plot representation of the mRNA levels showed that majority of CDK8 was expressed in varying degrees across the patient samples at medium to high levels, with the highest levels observed in CRC (Figure 1A). This was in line with a previous study that suggested a tumor promoting role for CDK8 in CRC. Moreover, increased CDK8 expression was significantly associated with decreased survival and clinic pathologic parameters of progression (Figures 1B,C). To confirm the results of the TCGA data, clinical samples of CRC were analyzed. The results showed that the expression level of CDK8 in the different clinical samples of CRC was higher than that in the corresponding normal tissues (Figures 1D,F). Furthermore, CDK8 expression in two CRC lines (HCT116 and LOVO) were also analyzed. The expression level of CDK8 was higher in the CRC lines, particularly in the HCT116 cells (Figures 1E,G). HIEC is a normal human intestinal epithelial cell line. Although HIEC is derived from intestine, it was widely used as the control for HCT116 and LOVO in a lot of studies (Wu et al., 2018; Hu et al., 2019). Our analysis identified CDK8 to be specifically overexpressed during CRC progression, highlighting its potential as novel therapeutic target in advanced CRC.

### Targeting CDK8 Sensitized CRC Cells to IR Through the Intrinsic Apoptotic Pathway

**FIGURE 2:**
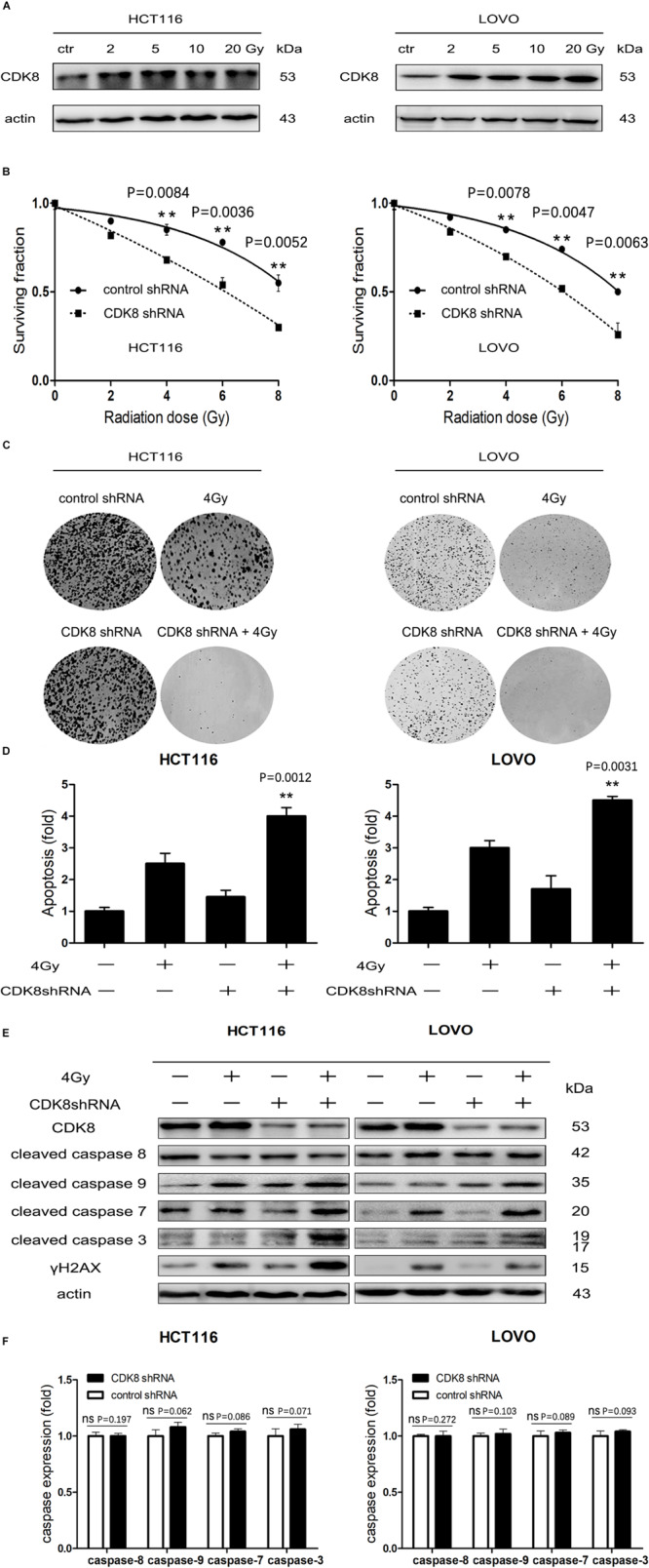
CDK8 Knockdown Increased IR-Induced Intrinsic Apoptotic Signaling in CRC Cells. **(A)** HCT116 (left) and LOVO cells (right) were irradiated with different doses of radiation and CDK8 protein level was analyzed by western blotting 24 h later. **(B)** HCT116 (left) and LOVO cells (right) transfected with CDK8 or control shRNA were exposed to different doses of radiation and cell viability was measured 24 h later. **(C)** Colony formation of HCT116 (left) and LOVO cells (right) transfected with CDK8 or control shRNA was assessed after irradiated with the indicated doses of radiation. **(D)** The apoptosis levels were assessed using Annexin V-FITC/PI double staining in CDK8 kncokdown HCT116 cells (left) and LOVO cells (right) compared with control shRNA cells at 24 h after 4 Gy irradiation. **(E)** The expression level of CDK8, γH2AX, cleaved caspase 9, cleaved caspase 8, cleaved caspase 7, and cleaved caspase 3 were detected by western blotting in CDK8 kncokdown HCT116 cells (left) and LOVO cells (right) compared with control shRNA cells at 24 h after 4 Gy irradiation. **(F)** The intensities of caspase-3,7,8,9 in CDK8 kncokdown HCT116 (left) and LOVO cells (right) were quantified after normalization to β-actin using Image J software. **p < 0.01. Typical results from three independent experiments are shown. The difference between two groups were analyzed with the Student’s *t* test. One-way analysis of variance (ANOVA) followed by *Post hoc* test was used in multiple groups.

We found that CDK8 was overexpressed and correlated with poor survival and tumor subtypes in CRC. However, it was not clear whether CDK8 participates in the IR response or affects CRC radiosensitivity. Therefore, we investigated the effects of IR on the expression of CDK8 in CRC. As shown in Figure 2A, CDK8 expression level was significantly increased in both HCT116 and LOVO cells following treatment with a series of IR doses. Furthermore, we explored whether decreased expression of CDK8 could enhance the radiosensitivity of CRC. We found that shRNA mediated knockdown of CDK8 when combined with IR, decreased cell viability and Colony formation in HCT116 and LOVO cells (Figures 2B,C). Analysis of the surviving fractions in HCT116 and LOVO cells showed that the survival fraction values at 4 Gy were reduced from 0.85 and 0.83 to 0.68 and 0.64, respectively.

To further understand the detailed mechanism of induction of radiosensitivity following CDK8 knockdown, we examined the apoptotic level and the expression of the apoptotic proteins. As shown in Figures 2D,E, the highest apoptotic level was detected and the apoptotic proteins of cleaved caspase 3, cleaved caspase 7, and cleaved caspase 9 but not cleaved caspase 8 were significantly increased in CDK8 knockdown CRC cells after IR treatment, suggesting that downregulation of CDK8 results in radiosensitivity primarily through the intrinsic apoptotic pathway. In addition, residual γH2AX, a sensitive and robust biomarker of DNA damage (Mah et al., 2010; Zhou et al., 2018), was significantly increased in CDK8 knockdown cells compared with control shRNA cells after IR treatment (Figure 2F).

### CDK8 Knockdown-Mediated Radiosensitivity of CRC Cells Was Dependent on e2f1 but Not p53

**FIGURE 3:**
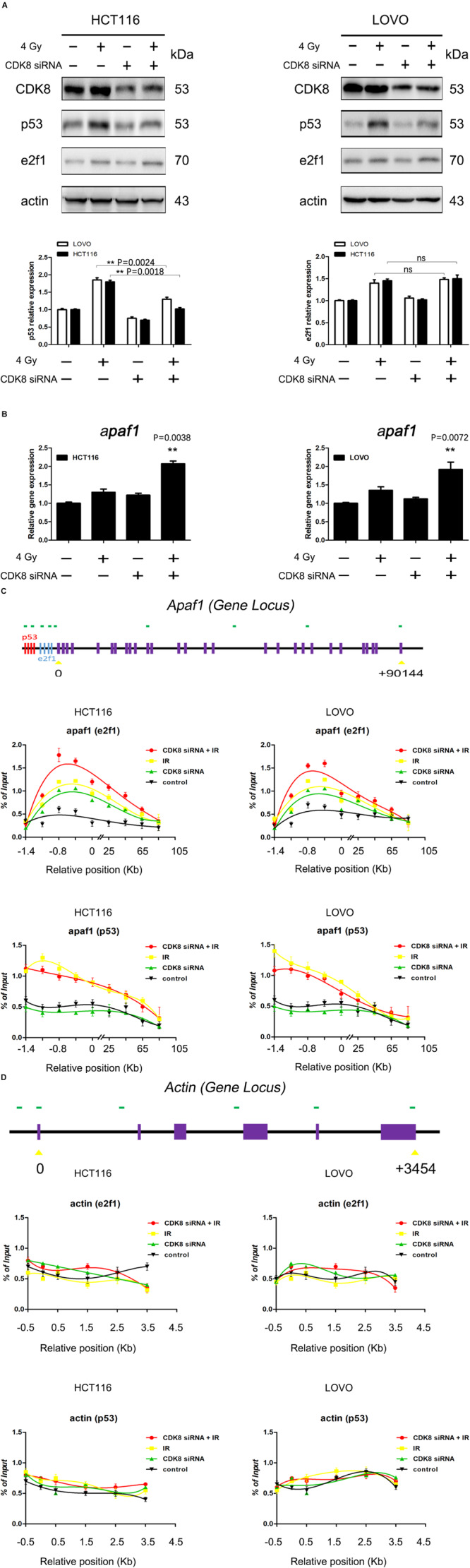
e2f1 But Not p53 Was Involved in IR-Induced Apoptosis in CDK8 Knockdown CRC Cells. **(A)** The expression of p53 and e2f1 were detected by western blotting in CDK8 kncokdown HCT116 cells (left) and LOVO cells (right) compared with control siRNA cells at 24 h after 4 Gy irradiation. The intensities of p53 and e2f1 (lower panels) were quantified after normalization to β-actin using Image J software. **(B)** apaf1 mRNA level in CDK8 knockdown HCT116 (left) and LOVO cells (right) compared with control siRNA cells were detected using qPCR analysis at 24 h after 4 Gy irradiation. **(C,D)** Schemes of the apaf1 **(C, top panel)** and housekeeping β-actin **(D, top panel)** gene loci. The positions of p53 and e2f1 binding sites in the apaf1 gene are shown. ChIP analyses to test for the binding of p53 and e2f1 at the apaf1 loci in the HCT116 **(C,D, left lower panels)** and LOVO **(C,D, right lower panels)** cell lines following the indicated treatments. **p < 0.01. Typical results from three independent experiments are shown. The difference between two groups were analyzed with the Student’s *t* test. One-way analysis of variance (ANOVA) followed by *Post hoc* test was used in multiple groups.

Our data provided compelling evidence that CDK8 regulated the radiosensitivity of CRC cells through the intrinsic apoptotic pathway. However, the potential regulatory mechanism underlying the activation of the intrinsic apoptotic pathway remained unclear. To elucidate the crosstalk between p53, e2f1, and CDK8 in IR-induced apoptotic signaling, the protein level of p53 and e2f1 was examined. As shown in Figure 3A, the expression of both p53 and e2f1 proteins were significantly increased in HCT116 and LOVO cells upon IR treatment. However, CDK8 knockdown inhibited the increased expression of p53 induced by IR, while e2f1 remained unaffected. The decreased level of p53 protein could be due either to decreased transcription of p53 gene regulated by CDK8, or to decreased p53 stability induced by CDK8-medaited phosphorylation.

Then, the expression levels of p53 and e2f1 target genes that link the apoptotic signaling pathways (Moroni et al., 2001; Lazzerini Denchi and Helin, 2005; Aubrey et al., 2018), including aen, noxa, p21, puma, bax, bcl2, fas, p73, mcl1, arf, bim, pkr, and apaf1 were analyzed. Our results showed that CDK8 knockdown combined with IR treatment failed to alter the mRNA levels of aen, noxa, p21, puma, bax, bcl2, fas, p73, mcl1, bim, pkr, and arf. By contrast, apaf1 gene expression was the most significantly increased after CDK8 knockdown together with IR treatment compared with IR treatment or CDK8 knockdown alone (Figure 3B and Supplementary Figure S1), indicating that the intrinsic apoptotic pathway induced by IR treatment in CDK8 knockdown CRC cells was triggered by apaf1.

To further assess the involvement of p53 and e2f1, we carried out a series of ChIP assays using cells that were untreated, or treated with CDK8 siRNA, 4 Gy, or both CDK8 siRNA and 4 Gy. The results of the ChIP analysis for apaf1 and the constitutively expressed housekeeping gene are shown in Figures 3C,D. As expected, IR treatment induced binding of p53 and e2f1 to the promoter region of apaf1, but not of the housekeeping gene. Importantly, in the CDK8 knockdown cells, there was a slight increase in the amount of e2f1 but not p53 associated with the apaf1 promoter, indicating that CDK8 served as a negative regulator of e2f1 transcription. Furthermore, e2f1 but not p53 was strongly recruited to the promoter region of apaf1 gene in irradiated CDK8 siRNA cells. Overall, these results demonstrated that the IR-induced intrinsic apoptotic pathway was mediated through potentiating transcription of apaf1 which is e2f1- and not p53-dependent.

### CDK8 Interfered With the Transcriptional Activity of e2f1 Independent of Mediator Complex

**FIGURE 4:**
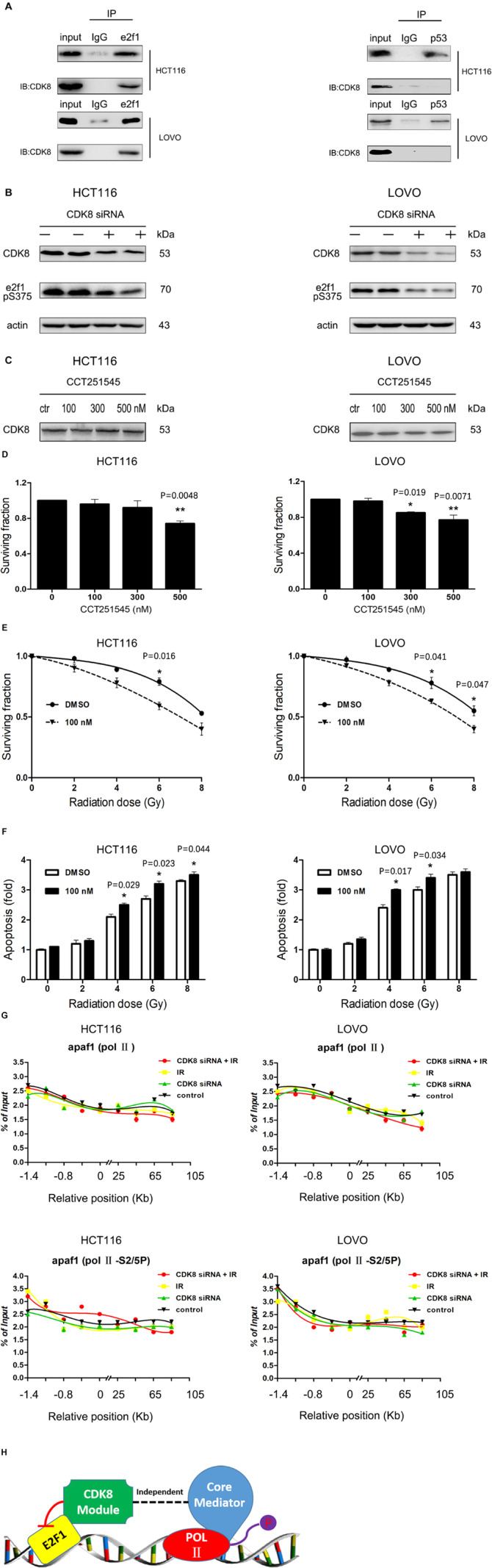
CDK8 Interfered with the Transcriptional Activity of e2f1 Independent of Mediator Complex. **(A)** Co-immunoprecipitation of CDK8 with e2f1 **(left)** or p53 **(right)** was performed with the whole cell extract of HCT116 or LOVO cells. **(B)** HCT116 **(left)** and LOVO cells **(right)** were treated with CDK8 siRNA, and the expression of CDK8, phosphorylated e2f1 (S375), and β-actin were assessed by western blotting. **(C)** HCT116 **(left)** and LOVO cells **(right)** were treated with various concentrations of CCT251545 for 24 h, and the expression of CDK8, phosphorylated e2f1 (S375), and β-actin were assessed by western blotting. **(D)** HCT116 **(left)** and LOVO cells **(right)** were treated with different doses of CCT251545 and the cell viability was measured 24 h later. **(E)** HCT116 **(left)** and LOVO cells **(right)** were pre-treated with 100 nM CCT251545 for 24 h prior to irradiation with different doses of radiation, and cell viability was measured 24 h after irradiation. **(F)** HCT116 **(left)** and LOVO cells **(right)** were pre-treated with 100 nM CCT251545 for 24 h prior to irradiation with different doses of radiation, and the apoptosis level was measured 24 h after irradiation. **(G)** ChIP analyses to test the effects of total Pol II **(top panels)** and Pol II S2/5P (center panels) on binding of apaf1 in HCT116 and LOVO cells. **(H)** Schematic illustration of the mechanism of co-regulation of e2f1 by CDK8. *p < 0.05 and **p < 0.01. Typical results from three independent experiments are shown. The difference between two groups were analyzed with the Student’s *t* test. One-way analysis of variance (ANOVA) followed by *Post hoc* test was used in multiple groups.

It has been reported that CDK8 interacts with and phosphorylates e2f1 in cells, and the phosphorylation represses activation of e2f1-dependent genes (Morris et al., 2008; Zhao et al., 2013). In this study, we further demonstrated that CDK8 interacted with e2f1 but not p53 in CRC cells using Co-IP methods (Figure 4A). Moreover, CDK8 knockdown decreased the phosphorylation of e2f1 (S375) (Figure 4B). In addition, using CCT251545, the most specific CDK8 inhibitor (Dale et al., 2015), we confirmed that the inhibition of CDK8 kinase activity leads to decreased e2f1 phosphorylation (Figure 4C), which was reported to be key for the increased transcriptional activity of e2f1. To further demonstrate the effect of CDK8 inhibitor as a radiosensitizer in CRC, cells were treated with 100 nM CCT251545 for 24 h prior to IR treatment. The results showed that CCT251545 sensitized CRC cells to IR treatment (Figures 4D–F). Therefore, the kinase activity of CDK8 is responsible for the efficacy of IR treatment in CRC.

Subsequently, we detected the role of CDK8 knockdown combined with IR treatment on the binding of RNA Pol II and its CTD phosphorylated forms S2/5P (which promotes Pol II detachment from the promoter and allows for the elongation of transcription). As shown in Figure 4G, the effect was less prominent for the binding of S2/5P and total Pol II on the apaf1 promoter, indicating that CDK8 regulated e2f1 transcriptional activity in a form independent of mediator.

Based on the above data, our studies revealed a detailed mechanism of CDK8 down regulating e2f1 transcriptional activity in a CDK8 kinase activity-dependent manner (Figure 4H).

### Targeting CDK8 Enhanced the Efficacy of Radiotherapy *in vivo*

**FIGURE 5:**
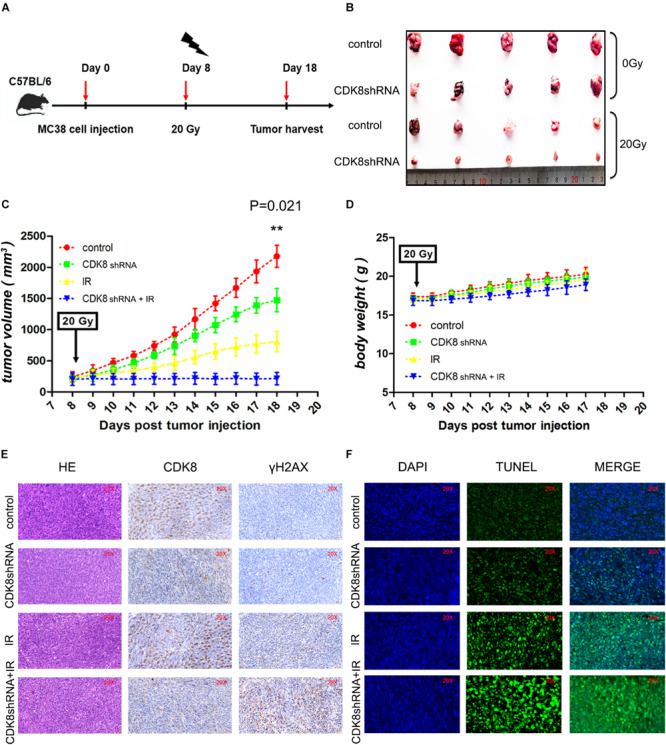
Targeting CDK8 Enhanced the Efficacy of Radiotherapy *in vivo*. **(A)** A schematic of the experimental schedule was shown. **(B)** Each group of mice was composed of five C57BL/6 wild type female mice. Control vector MC38 cells (control, 1 × 10^7^ cells) and CDK8 knockdown MC38 cells (CDK8 shRNA, 1 × 10^7^ cells) were inoculated under the dorsal skin of the mice. After 8 days of injection, the treated mice were irradiated with 0 Gy or 20 Gy X ray. The mice were sacrificed 18 day after injection, and tumors were excised. **(C,D)** The volumes **(C)** and net weights **(D)** of the tumors in each group are shown. **(E)** CDK8 and γH2AX protein expression was verified by IHC staining. Necrotic cells in tumor sections were visualized by H&E staining. **(F)** Apoptotic cells (green) in the tumor sections were identified by TUNEL assay and the nuclei were counterstained with DAPI (blue). **p < 0.01. Typical results from three independent experiments are shown. The difference between two groups were analyzed with the Student’s *t* test. One-way analysis of variance (ANOVA) followed by *Post hoc* test was used in multiple groups.

To determine whether downregulation of CDK8 can sensitize CRC to IR treatment *in vivo*, MC38 with reduced CDK8 expression were injected into C57BL/6 wild type mice. As shown in Figures 5A–D, reducing CDK8 expression significantly increased the inhibitory effects of 20 Gy on tumor growth. The volumes of tumors from the group with CDK8 shRNA combined with IR treatment were significantly decreased, indicating that knockdown CDK8 increases the radiosensitivity of CRC *in vivo*. In addition, immunohistochemical staining verified the expression of CDK8 protein and positive staining for γH2AX in the combination treatment (Figure 5E). To examine whether the radiosensitivity mediated by CDK8 *in vivo* was the result of apoptosis, we performed TUNEL assays. As shown in Figure 5F, the apoptotic cell population in CDK8 shRNA combined with IR treatment was significantly greater than that in IR treatment alone, indicating that IR synergized with CDK8 shRNA to enhance IR induced apoptosis *in vivo*. These results suggest that CDK8 knockdown improves the radiosensitivity of CRC *in vivo* through the apoptotic pathway, which is consistent with the *in vitro* data described above.

### Clinical Drug Ponatinib Increased Sensitivity of CRC Cells to IR

**FIGURE 6:**
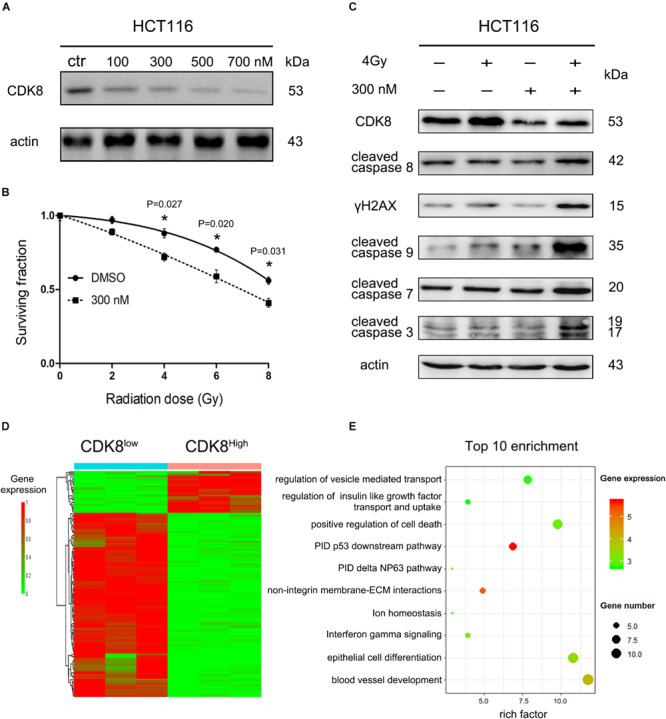
Clinical Drug Ponatinib Increased Radiosensitivity of CRC Cells. **(A)** HCT116 cells were treated with different doses of ponatinib and the protein level of CDK8 was analyzed by western blotting 24 h later. **(B)** HCT116 cells were pre-treated with 300 nM ponatinib for 24 h prior to irradiation with different doses of radiation, and cell viability was measured after 24 h. **(C)** HCT116 cells were pre-treated with 300 nM ponatinib for 24 h before 4 Gy irradiation, and the expression level of CDK8, γH2AX, cleaved caspase 9, cleaved caspase 8, cleaved caspase 7, and cleaved caspase 3 were assessed by western blotting after 24 h. **(D)** Heatmap shows the 122 differentially expressed genes (DEGs) in CDK8^Low^ versus CDK8^High^. Among the 122 genes, 28 were downregulated and 94 were upregulated. **(E)** A bubble plot displays the 10 most significant terms identified by Metascape analysis. Bubble colors represent the corrected *P* values. *p < 0.05. Typical results from three independent experiments are shown. The difference between two groups were analyzed with the Student’s *t* test. One-way analysis of variance (ANOVA) followed by *Post hoc* test was used in multiple groups.

The role of CDK8 in the radiosensitivity of CRC highlights the potential application of CDK8 inhibitors in IR therapy. To explore the potential clinical application of CDK8 inhibitors, biochemical screening of the available clinical and preclinical kinase inhibitors from The IUPHAR/BPS Guide to PHARMACOLOGY^1^ revealed potent binding activity for ponatinib (Dale et al., 2015; Supplementary Figure S2A). According to our experimental data (Figure 6A), the expression level of CDK8 was significantly decreased after treating with different concentrations of ponatinib. Our results also showed that ponatinib combined with IR treatment synergistically decreased cell viability in HCT116 cells (Supplementary Figure S2B and Figure 6B). Concomitantly, high apoptotic level (Supplementary Figures S2C,D), and proteins expression level of cleaved caspase 3, cleaved caspase 7, cleaved caspase 8, cleaved caspase 9, and γH2AX was detected (Figure 6C).

To investigate the immediate changes in gene transcription in response to CDK8 inhibition and IR treatment, we conducted RNA-seq analysis. As shown in Figure 6D and Supplementary Figure S2E, after IR treatment, the majority of the differentially expressed genes associated with low CDK8 levels are upregulated compared to their expression in the high CDK8 group, and metascape analysis used to evaluate the biological functions of the 94 upregulated genes showed the top 10 clusters of enriched sets. These genes were significantly associated with the pathway interaction database (PID) P53 downstream pathway (Figure 6E), which contains e2f1 and apaf1. These results further demonstrate the core role of CDK8 in radiosensitivity of CRC. Overall, our data demonstrate the potential clinical application of the CDK8 inhibitor.

## Discussion

The role of CDK8 as an oncogene has been increasingly recognized and widely reported in several human cancers (Bragelmann et al., 2017; Roninson et al., 2019). To determine the expression pattern of CDK8 in human cancers, we performed a pan cancer analysis of CDK8 transcriptome profiles. Analysis of TCGA RNA expression data showed that CDK8 expression was highest in CRC, and associated with significantly decreased disease-free survival (Figures 1A–C). The identification of cancer types with frequent amplification of CDK8 and correlation of expression with survival suggests a potential role of CDK8 in the pathogenesis of CRC or its treatment response, but it does not necessarily imply that such cancers will respond to CDK8 inhibitor therapy. Our study provides evidence that inhibiting CDK8 activity genetically or pharmacologically affects CRC growth, and when combined with IR treatment, it significantly augments IR sensitivity of CRC *in vitro* and *in vivo*.

Apoptosis is thought to be the major mechanism of IR-induced cell death and aberrant apoptotic signaling can cause resistance to radiotherapy (Baskar et al., 2012; Cao et al., 2019). It was reported that IR causes DNA damage that activates p53 and e2f1 pathways in concert or independently to repair the damage or induce apoptosis (Udayakumar et al., 2010). In our study, the protein expression of p53 were downregulated by IR treatment when combined with CDK8 knockdown (Figure 3A). This may be attributed to the role of CDK8 as a positive regulator of p53-dependent transcription (Donner et al., 2007), and this effect was blocked when CDK8 expression was reduced. Therefore, one possible scenario is that a e2f1-mediated alternative pathway that is p53 independent plays an essential role. As expected, our results showed that both p53 and e2f1 induced an increase in apaf1 level following IR treatment, however, the preferential transcriptional activation of apaf1 in CDK8 knockdown CRC cells prior to IR was regulated by e2f1 and not p53 (Figure 3C). This, along with the fact that e2f1 is a more potent inducer of apoptosis in cancer cells, suggests that the increase in apaf1 level is a significant event in e2f1-induced apoptosis in CDK8 knockdown CRC cells.

Transcriptional regulation is considered to be a critical determinant of gene expression, which is a complex process requiring the concerted action of numerous transcription factors and transcriptional co-factors (Lee and Young, 2013; Maeshima et al., 2015). As a nuclear serine threonine kinase, CDK8 has been associated with both positive (Galbraith et al., 2010) and negative (Elmlund et al., 2006) regulatory roles in transcription through mechanisms that include regulation of transcription factor turnover, regulation of CTD phosphorylation, and regulation of activator or repressor function. Previous studies reported that CDK8 physically interacts with e2f1, and is a conserved negative regulator of e2f1-dependent transcription (Morris et al., 2008; Zhao et al., 2013). In our current study, ChIP analysis revealed that CDK8 knockdown enhanced the recruitment of e2f1 to the promoter of the e2f1-responsive gene apaf1 (Figure 3C). This effect of CDK8 inhibition is specific to e2f1-induced genes and not with constitutively expressed gene such as actin (Figure 3D). Importantly, following CDK8 knockdown, there was already a slight increase in the amount of e2f1 associated with the promoters. Thus, CDK8 inhibition could potentiate the basal transcriptional effects of e2f1, and continuously enhance apaf1 transcriptional activity under external stimulation, such as with IR (Figure 3B). A high concentration of apaf1 may increase the probability of physical interaction between procaspase-9 molecules, resulting in oligomerization and subsequent autoactivation of procaspase-9. Then activated caspase-9, in turn, cleaves and activates downstream caspases-3/7, effector proteases that execute the cell death program (Figure 2E). In short, we propose that CDK8 knockdown enhanced the transcriptional activity of e2f1 leading to increased apaf1 level which subsequently triggered the downstream apoptosis cascade and finally induced mitochondria-dependent intrinsic apoptosis to enhance radiosensitizing effect in CRC.

Notably, it has been hypothesized that the negative effect of the CDK8 module on eukaryotic transcription is mediated by CDK8-dependent phosphorylation of CTD (Elmlund et al., 2006). The hyper phosphorylated form of CTD binds less tightly to the mediator, which may result in the dissociation of pol II from the holoenzyme complex (Eick and Geyer, 2013). However, we analyzed the effects of CDK8 knockdown combined with IR on the binding of total RNA Pol II and its CTD phosphorylated forms, S5P and S2P, but did not find any increase in the association of any of the forms of Pol II with the promoter or any other part of the e2f1 responsive gene, apaf1 (Figure 4G), indicating that Pol II CTD phosphorylation in the context of activated genes seems to be independent of the mechanism of transcriptional regulation of e2f1 by CDK8. This might be attributed to the fact that although majority of CDK8 sub complex appears to be associated with various forms of mediator in human cells, up to 30% of CDK8 may exist in a form independent of the mediator (Knuesel et al., 2009a, b). It has been reported that the phosphorylation of S375 in e2f1 by CDK8 may be a general mechanism of regulation of e2f1 transcriptional activity (Zhao et al., 2013). This was mirrored by the CDK8 kinase inhibitor, which prevented the phosphorylation of e2f1 and increased the radiosensitivitty in both HCT116 and LOVO cells (Figures 4D–F). In general, our studies revealed a detailed mechanism of CDK8 mediated downregulation of e2f1 transcriptional activity in a CDK8 kinase activity -dependent manner independent of the mediator.

γH2AX is a sensitive and robust biomarker of DNA damage (Xiao et al., 2018). Several studies showed that IR enhanced γH2AX also via apoptotic signals (Solier and Pommier, 2014). For example, IR caused the release of mitochondrial cytochrome c into the cytoplasm to activate caspase-3, which triggers the apoptotic cascade to DNA fragmentation including phosphorylation of H2AX (Harada et al., 2014). In addition, DNA repair is curtailed because of caspase-mediated cleavage and inactivation of key DDR (DNA damage response) mediators (such as MDC1) (Dimitrova and de Lange, 2006) and effectors (such as PARP1) (Lazebnik et al., 1994). Along the same lines, caspase-3 also inactivates DNA replication by cleaving CDC6 (Yim et al., 2006). In the present study, we found that targeting CDK8 selectively radiosensitized colorectal cancer through the mitochondria dependent intrinsic apoptotic signaling, which triggered by caspase-3, 7, 9. Besides, the increase of γH2AX was also significant in CDK8 knockdown CRC cells after IR (Figures 2E, 5E). This prompted us to hypothesize that CDK8 knockdown enhanced the transcriptional activity of e2f1 leading to increased apaf1 level, which homo-oligomerizes into a caspase-activating complex that sequentially recruits and activates the initiator caspase-9 and the effector caspases-3/7. Caspases activation in those cells could lead to downstream nuclease activation and DNA fragmentation, which subsequently induced significantly higher levels of γH2AX.

**FIGURE 7:**
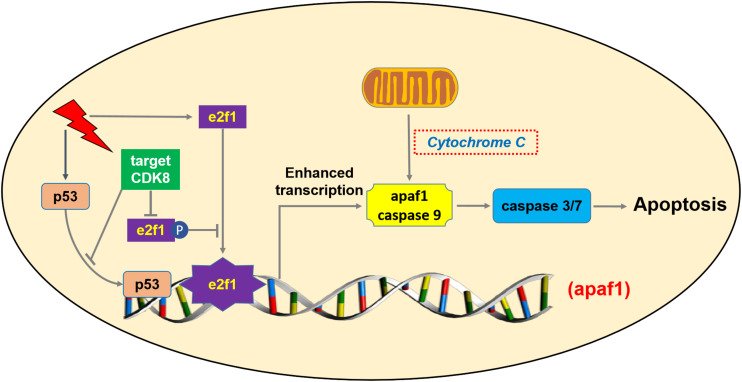
Schematic sepresentation of the mechanism of CDK8 mediated radiosensitization of CRC cells through the mitochondria-dependent intrinsic apoptotic signaling through e2f1-dependent induction of apaf1.

Based on the data presented above, amplification of CDK8 may be a novel way for cells to overcome regulation by e2f1, allowing cells to prevent the expression of e2f1 target genes that are pro-apoptotic. By abrogating e2f1 activity, CDK8 appears to promote cell proliferation at least in CRC. CDK8 mediated regulation of e2f1 appears to be a general mechanism to achieve control over e2f1 mediated activation or repression of transcription. Potentially, targeting CDK8 for inhibition, at least in CDK8 over-expressing tumors, could help restore e2f1 activity in these tumors, promoting inhibition or regression of tumor growth. In summary, our study demonstrated that knockdown or inhibition of CDK8 can inhibit the fractional survival of CRC cells, and targeting CDK8 increased IR-induced apoptosis *in vivo* and *in vitro*. Increased level of apaf1 resulting from enhanced transcriptional activity of e2f1 (not p53) could provide an explanation for the greater sensitivity of CRC cells to IR following CDK8 knockdown (Figure 7). Thus, CDK8 is a potent candidate target to enhance cancer radiotherapy.

## Data Availability Statement

All datasets generated for this study are included in the article/Supplementary Material. Any material described in the article can be requested directly from the corresponding author, without undue reservation, to any qualified researcher.

## Ethics Statement

All animal experiments procedures and uses of clinical samples were performed according to guidelines approved by Committee review of animal experiments in Anhui Medical University. Written informed consent for publication was obtained from all participants.

## Author Contributions

GZ and BC conceived of the study and wrote the manuscript. BC, GH, YG, PW, KZ, and XQ conducted the experiments. SC, AX, and LW analyzed and interpreted the data. All authors read and approved the final manuscript.

## Conflict of Interest

The authors declare that the research was conducted in the absence of any commercial or financial relationships that could be construed as a potential conflict of interest.

## Footnotes

1 https://www.guidetopharmacology.org
