# Supplementary material for: Antagonizing CDK8 Sensitizes Colorectal Cancer to Radiation Through Potentiating the Transcription of e2f1 Target Gene apaf1
Source: Front Cell Dev Biol. 2020 Jun 12;8:408. doi: 10.3389/fcell.2020.00408 (PMC7304162; doi:10.3389/fcell.2020.00408)
Supplement: Supplementary file 1 [file Data_Sheet_1.pdf]

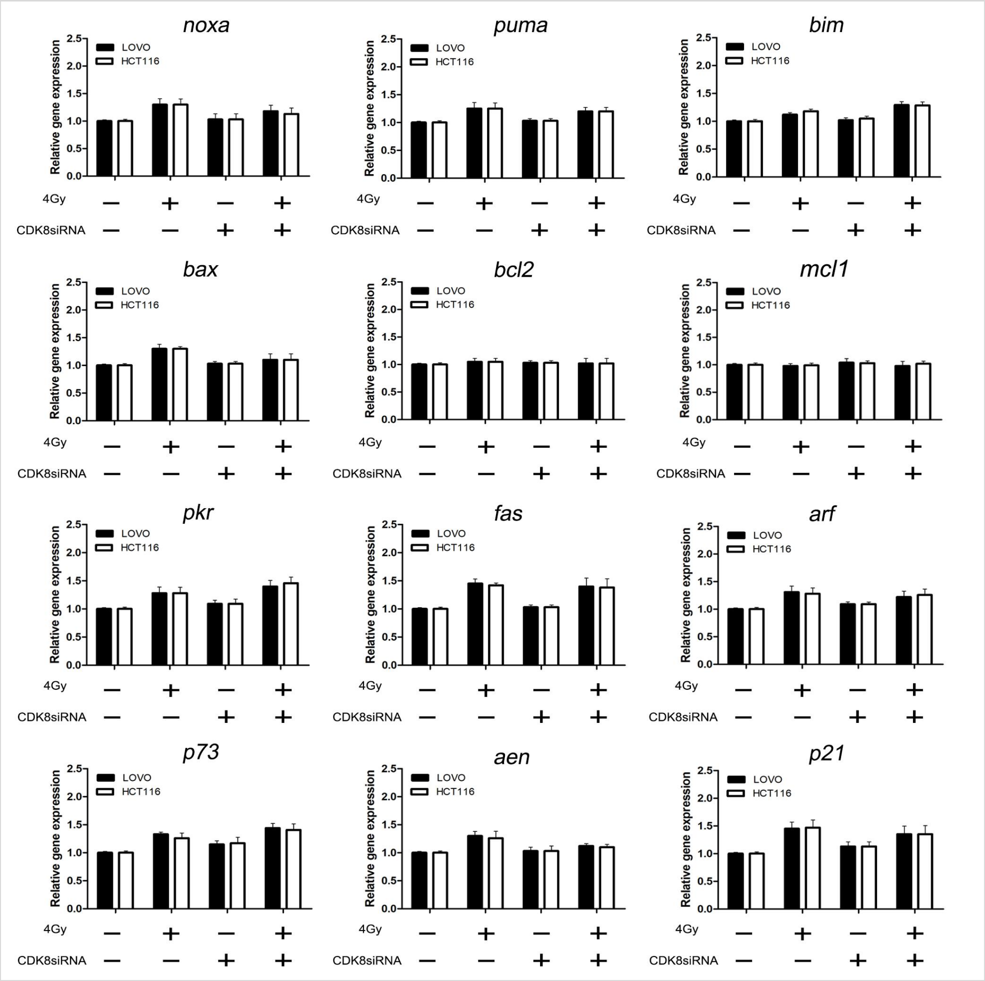

FIGURE S1. Effect of CDK8 Knockdown Together with IR Treatment on the Expression of p53 and e2f1 Target Genes that Link the Apoptotic Signaling Pathways

The mRNA level of aen, noxa, p21, puma, bax, bcl2, fas, p73, mcl1, arf, bim, and pkr was assessed using qPCR analysis in CDK8 knockdown HCT116 (white) and LOVO cells (black) compared with control siRNA cells 24 h after 4 Gy irradiation. Typical results from three independent experiments are shown. The difference between two groups were analyzed with the Student's t test. One-way analysis of variance (ANOVA) followed by Post hoc test was used in multiple groups.

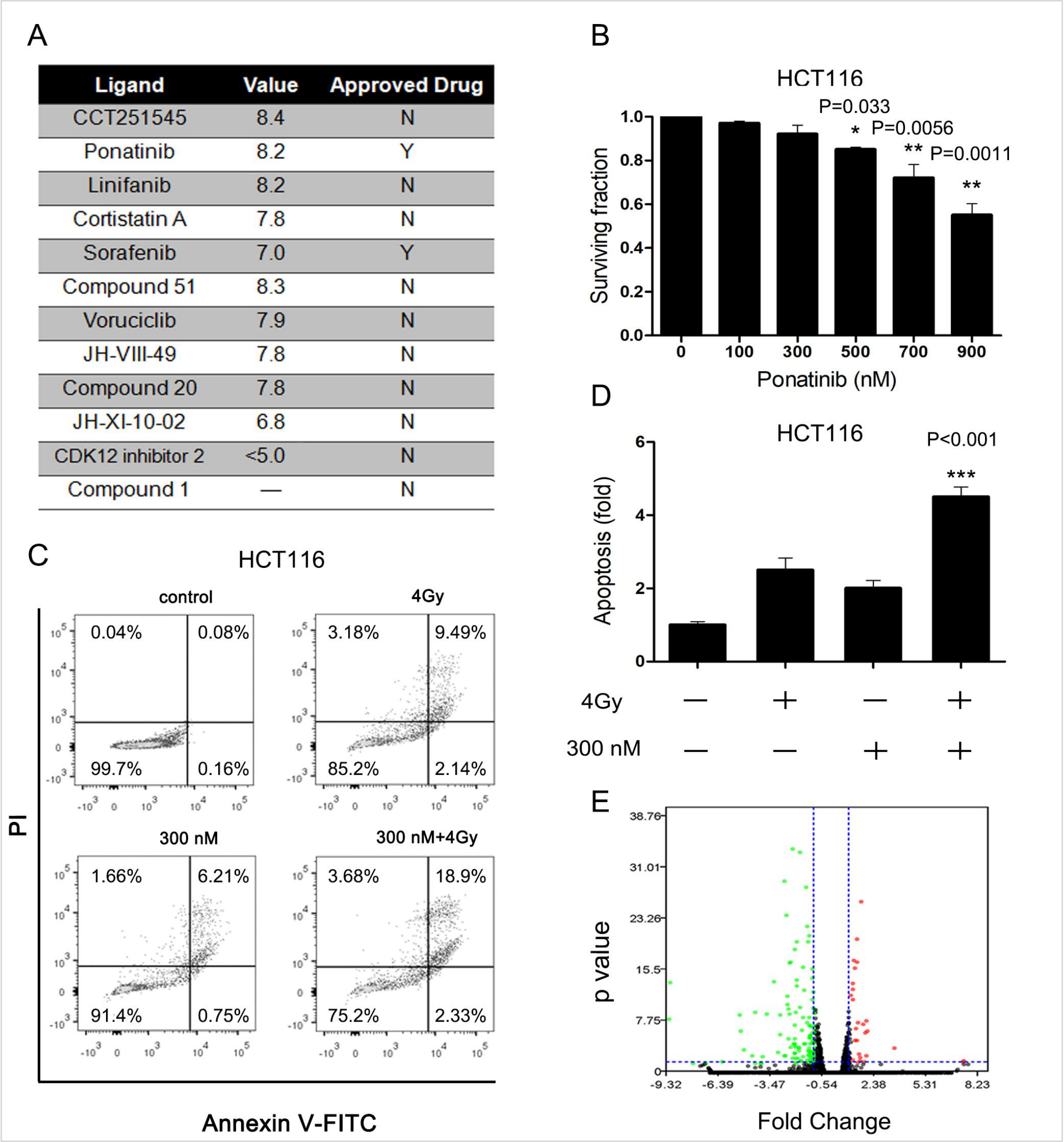

Ligand	Value	Approved Drug
CCT251545	8.4	N
Ponatinib	8.2	Y
Linifanib	8.2	N
Cortistatin A	7.8	N
Sorafenib	7.0	Y
Compound 51	8.3	N
Voruciclib	7.9	N
JH-VIII-49	7.8	N
Compound 20	7.8	N
JH-XI-10-02	6.8	N
CDK12 inhibitor 2	<5.0	N
Compound 1	—	N

FIGURE S2. Clinical Drug Ponatinib Augmented IR Sensitivity of CRC

(A) CDK8 inhibitors given by The IUPHAR/BPS Guide to PHARMACOLOGY. (B) HCT116 cells were treated with different doses of ponatinib, and cell viability was measured 24 h later. (C) HCT116 cells were pre-treated with 300 nM ponatinib for 24 h before 4 Gy irradiation, and apoptosis levels were assessed using Annexin V-FITC/PI double staining at 24 h after irradiation. (D) Quantification of apoptosis levels in (C). (E) Volcano plot showed the 122 DEGs in control versus radiation combined with drug treatment. Low expression was presented in green, and high expression was presented in red. *p<0.05, **p<0.01 and ***

p<0.001. Typical results from three independent experiments are shown. One-way analysis of variance (ANOVA) followed by Post hoc test was used in multiple groups.

TABLE S1. siRNA and shRNA Sequences of CDK8

Gene	Target Sequence
CDK8 siRNA #1 (Homo sapiens)	CCTCTGGCATATAATCAAGTT
CDK8 shRNA #1 (Homo sapiens)	GAATGGTGAAGTCACTATTAT
CDK8 shRNA #2 (Mus musculus)	GCAATTGATATTTGGGCTATA

TABLE S2. Primer Sequences for ChIP Analysis of actin

Amplicon Site	Forward Primer	Reverse Primer
-421 to -559	TGCGCATAGCAGACATACAAC	GCTTTTGAGGGTAACAGTCACG
24 to 156	GCGAGCACAGAGCCTCGCC	CTCTGCACGGGCGAAGGGGCC
611 to 708	CGCTTGCGCGCACTTCCT	GCCTCCGCCCGGTTCAAACAG
1754 to 1860	GAACAGACTCCCCATCCCAAG	GTCACACTGGGGAAGCCACT
2604 to 2729	TGTCTTTCCTGCCTGAGCTG	GCTCAGGAGGAGCAATGATC
3295 to 3420	TTGAATGATGAGCCTTCGTG	CTCAAGTCAGTGTACAGGT

TABLE S3. Primer Sequences for ChIP Analysis of apaf1

Amplicon Sites	Forward Primer	Reverse Primer
-222 to -338	CGGTTTATAACAACGTGAACGC	CTCAGAAGGAGTTGAGGCTAAG
-529 to -649	AGGCCGGCCCAGGCAGCCTC	GTGGCCCGGAGCAGCGGAGG
-692 to -821	AGGGGCTCCCTTGGGCCCCGAC	CGCCAAGGCGGCCACGTCCTGC
-1179 to -1340	ACTTCTACGCGCGCGGGCATG	CTTCTCCGCCTCTGGACAGCGG
-1345 to -1461	GGTAGCGAGTGGACGTGACTGC	CTACTCCGGATGCCGCTGC
25094 to 25209	GTGCTCAGCCCCAGCTAATATTC	CAGATGGCCAAAATCTCACCGG

45112 to 45195	CTGGGTAGAATTCTACTTTG	CCACTTACCTGAATATTCTACC
65600 to 65674	GTAAAGCTGTAGAGCTCAGC	GAACTTACACCTAGGTCTCC
89985 to 90079	GAGATACCTAGTATGATGG	GAAATGCTCATCTGCAAACTAC

TABLE S4. Primer Sequences for RT-PCR Analysis

Gene	Forward Primer	Reverse Primer
actin	GGACCTGACTGACTACCTCAT	CGTAGCACAGCTTCTCCTTAAT
aen	AGGGCTGTTGGTTCTTTCTT	TGTTGGCACATTCCCTACTC
apaf1	GGACGACAGCCATTTCCTAATA	GCAGCTTAGCTTGCTGATAAAC
arf	AGCTGTCGACTTCATGACAAG	GAGCTTTGGTTCTGCCATTTG
bax	TCTGACCCTAGCTCTTTCCT	CACCTGTAATCCCAGCACTT
bcl2	TAGTGTGTATGCCCTGCTTTC	CCTCTGTGATGCTGAAAGGTTA
bim	CTCTGTGCCTGCTCCTTATG	CCCTAACCTGACTCTTTCTTGG
fas	GGGAAGGAGTACACAGACAAAG	GGTCCGGGTGCAGTTTATT
mcl1	GTGAAGATGGTAGGGTGGAAAG	TCGGCGGGTAATCAATTCTATG
noxa	GAAGGGAGATGACCTGTGATTAG	TGCTGAGTTGGCACTGAAA
p21	CTTGTCCTTTCCCTTCAGTACC	TTCTTCTTGTGTGTCCCTTCC
p73	AATCTCTCGCAGTATGTGGATG	GTTGTACAGGATGGTGGTGAA
pkr	AGAACACTGCACTGAAGATAGG	GGATGGCCGTTTAGAGAGAAA
puma	TCGGTGCTCCTTCACTCT	CGTTTGGCTCATTTGCTCTTC
